# AI in interventional cardiology: Innovations and challenges

**DOI:** 10.1016/j.heliyon.2024.e36691

**Published:** 2024-08-26

**Authors:** Dmitrii Khelimskii, Aram Badoyan, Oleg Krymcov, Aleksey Baranov, Serezha Manukian, Mikhail Lazarev

**Affiliations:** a Meshalkin National Medical Research Center, Ministry of Health of Russian Federation, Novosibirsk, Russian Federation; b HSE University, Russian Federation

## Abstract

Artificial Intelligence (AI) permeates all areas of our lives. Even now, we all use AI algorithms in our daily activities, and medicine is no exception. The potential of AI technology is hard to overestimate; AI has already proven its effectiveness in many fields of science and technology. A vast number of methods have been proposed and are being implemented in various areas of medicine, including interventional cardiology. A hallmark of this discipline is the extensive use of visualization techniques not only for diagnosis but also for the treatment of patients with coronary heart disease. The implementation of instrumental AI will reduce costs, in a broad sense. In this article, we provide an overview of AI research in interventional cardiology, practical applications, as well as the problems hindering the widespread use of neural network technologies in interventional cardiology.

## 1 Introduction

Interventional cardiology is a complex field of medicine that focuses on the diagnosis and management of cardiovascular diseases. A hallmark of interventional cardiology is the extensive use of multiple visualization techniques not only for diagnosis but also for the treatment of patients with cardiovascular diseases. These patients presented a wide range of pathologies, such as coronary artery disease, heart failure, arrhythmia, and valvular heart disease, that require a personalized approach to treatment, making it challenging to implement effective therapeutic and preventive measures [1]. It is important to recognize that even the most effective treatment methods may yield varying results at an individual level [2]. Based on this, large datasets can serve as the foundation of personalized medicine, where Machine Learning (ML) algorithms predict individual patient risks and accurately determine the application points for specific treatment methods. The multidimensional data extracted through such technologies, combined with the potential for real-time, two-way interaction between patients and healthcare providers, hold the promise of delivering more detailed and dynamic personalized care [3], [4], [5], [6]. Despite the great potential, the impact of AI in interventional cardiology is still limited. The objective of this article is to demonstrate the widespread use of ML and the potential of artificial Intelligence (AI) in interventional cardiology, as well as to provide insights into the prospects and future directions of this scientific field.

## 2 Background for artificial intelligence development

In the present stage of scientific advancement, there is a debate surrounding the efficacy of the traditional paradigm, which involves conducting large-scale studies on specific pathologies [6]. According to several authors [7] this approach is considered inefficient in addressing cardiovascular diseases. Given this, there is a growing need for the implementation of new research modalities capable of economically analyzing large volumes of information. Special attention is given to ML methods on big data, as they have the potential to improve diagnostic and treatment outcomes with lower costs [8], [9], [10]. Since the creation of the first electronic computer in the mid-20th century until the present day, there has been a continuous integration of electronic programmable devices into all aspects of human life. The field of medicine is not exempt from this trend. As a result of modernizing the workflow - there has been widespread implementation of advanced technologies, ranging from the routine use of computers in workstations and electronic medical charts to the performance of fully robotic surgeries [11]. An important aspect of this globalization has been the ability to store large volumes of information compactly and conveniently [12]. The translation of document management into electronic format and the structuring of data have contributed to the creation of extensive databases on specific diseases, treatment methods, and global statistical indicators [13]. The adoption of electronic health records, increased accessibility of digital medical data from emerging applications [14], biosensors [15], and the advancement of communication technologies have facilitated the accumulation of large data sets available for analysis and synthesis [16]. The constantly expanding volumes of data necessitate the use of more efficient methods for processing them in order to practically apply the knowledge gained. With this goal in mind, in recent years, the capabilities of AI have been introduced into the field of medicine [17]. The most crucial areas of AI application in healthcare include: automatic speech recognition and natural language processing [18]; predictive, recommendation, and diagnostic algorithms [19]; computer vision and image analysis [20]; and robotics [21]. While automatic speech recognition is already used in medicine [22] to record clinical patient information, the use of prediction and recommendation algorithms is of particular interest, especially to practicing physicians. This will allow automating repetitive tasks, such as evaluating diagnostic tests, and acquiring practically significant knowledge through the analysis of clinical data [23]. Thus, the application of ML is a new branch in the field of innovative medicine that is increasingly being used in clinical research to improve prognostic modeling and identify new predictors of adverse outcomes.

## 3 Statistical and machine learning methods

The concept of big data emerged back in the 1990s, and yet, a precise definition and universally accepted threshold for big data do not exist. This term refers to data sets that are too large or complex for traditional statistics. Statistical data analysis is actively used in medicine. One of the strongest advantages of this approach compared to others is its relative simplicity and interpretability. However, often big data research cannot be described by simple models due to intricate dependencies and vast dimensionality. To provide an idea of the scale of such data, consider a database comprising 100,000 patients with 100 attributes. Beyond size, the challenge with big data lies in its complexity, which is based on heterogeneity, high dimensionality, and the fact that it is dynamic (all previous separate measurements are dynamically connected). The most popular description of big data was proposed by D. Laney in 2001 and is known in the academic world as the “3Vs”: volume, variety, and velocity [24].

The subsequent development phase in medical science allowed algorithms to learn autonomously and make clinical decisions. The sheer volume of data becomes an obstacle for its manual processing by humans, and at a certain point impacted the development of medical science. This resulted in an increased use of artificial intelligence algorithms for data analysis. AI is defined as the theory and development of computer programs and systems capable of performing tasks requiring human-level intelligence [3], [23]. ML is a subset of AI that uses algorithms autonomously deriving knowledge by extracting patterns from data [25]. ML is an algorithm capable of iteratively discerning patterns (learning) to optimize forecasting or classification tasks [3].

All ML algorithms are based on the minimization of a function called the loss function. The loss function is a measure of how poor a prediction model is in terms of its ability to predict the expected outcome, or in other words, it's a measure of error.

The particular structure of the loss function can be quite diverse, but the most common ones are $loss_{L_{2}}=\frac{1}{N}\sum_{N}{\left(y-\overset{ˆ}{y}\right)}^{2}$ and $loss_{L_{1}}=\frac{1}{N}\sum_{N}|(y-\overset{ˆ}{y})|$ where y is algorithm's output, and $\overset{ˆ}{y}$ is true value. By minimizing the error step by step, one can improve the model using functional optimization methods. One of the most common classes of methods is gradient descent methods. One of the earliest and still widely used optimization methods is the Stochastic Gradient Descent (SGD) [26]. The essence of the method involves iteratively differentiating the loss function and step by step adjusting the algorithm's parameters. Today, there are many different variations of optimization methods from this class, but the most popular is Adam [27].

ML methods address a wide range of tasks, from diagnosing using tabular data to noise reduction or data imputation. Below, we will introduce several of the most common ML methods for labeled data (supervised learning).

**Linear and Logistic Regression**

Linear models [32] are widely used in practice for solving classification and regression problems. Linear models make predictions using a linear function of input features: $Y=a_{0}+a_{1}x_{1}+...+a_{N}x_{N}$ where Y is the target value, $x_{N}$ are the features, and $a_{N}$ are the coefficients.

Fig. 2 (a) depicts an abstract example of the final optimized line with the least error from one feature; it should be mentioned that features can be nonlinear functions. Linear regression is a simple and easily interpretable method, often used when a numerical prediction is needed [33][34]. For classification tasks where one needs to predict the probability of an object belonging to one class or another based on its features, logistic regression is employed.

Logistic Regression [35] is used for modeling the probability of the existence of a certain class or event, such as male/female or healthy/sick (the number of classes is not limited), based on a set of feature values. In classification tasks, it is necessary to estimate the probability of various outcomes. In such cases, logistic regression is used, where the result of the regression is the probability of outcomes or, in other words, the likelihood of a given object belonging to one or another class. Logistic regression is applied as one of the tools for statistical analysis in various scientific fields, particularly in medicine [36], and is also frequently encountered alongside other methods. Due to its nature logistic regression assumes a linear relationship between the features and the target variable which makes it hard to capture non-linear dependencies. To overcome this drawback one of the most popular ML methods can be helpful: decision trees.

**Figure 1:**
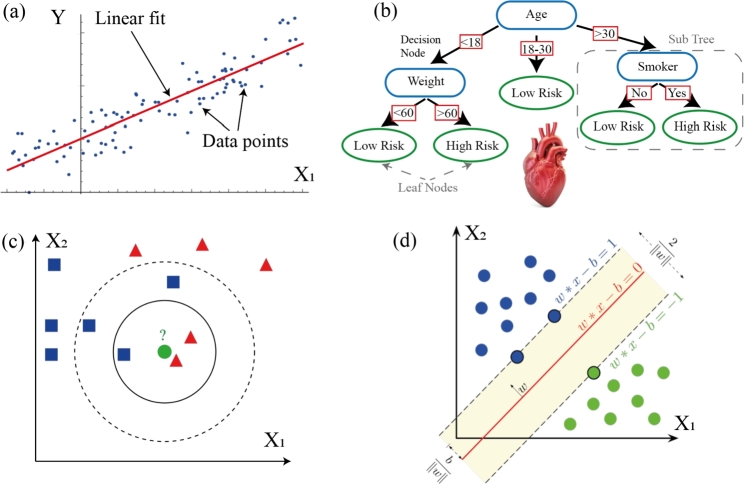
Schematic illustration of ML algorithms. (a) Linear regression illustration example taken from [28] (b) Decision trees taken from [29] (c) k-NN taken from [30](d) Method of Support Vector Machines taken from [31].

**Decision trees** are well-suited for working with tabular data [37]. There are many different decision tree algorithms, and they remain popular and continue to evolve. An abstract algorithm is shown in Fig. 1 (b), where each node of the tree contains a certain feature and a criterion that determines further descent down the tree to the final prediction result. Overfitting is a major problem for deep trees. It was suggested that instead of one deep tree, an ensemble of trees should be used. An example of such an ensemble method is the random forest method [38]. The algorithm involves creating N random subsets of the data sample, and then a decision tree is trained for each subset. The final model is the result averaged over the N trained trees. Another class of methods based on decision trees is called gradient boosting [39]. Gradient boosting is an iterative method, where a tree is constructed at each iteration to correct the results of the previous one. This method shows some of the best results on tabular data and is widely used, achieving state-of-the-art (SOTA) results [40]. There are also various libraries and its subtypes available [41].

**k-nearest neighbors algorithm** is a classic ML method for data clustering [42]. The essence of the method is quite simple: we look at the K nearest neighbors in feature space, as schematically shown in Fig. 1 (c), and determine the class of the object according to the majority class represented in the K nearest neighbors. k-NN is widely used in classification tasks but has several drawbacks, the most significant of which is a strong sensitivity to hyperparameters, such as the number of nearest neighbors and computational complexity. Nevertheless, it is frequently used due to interpretability capabilities. An example of its use in medicine can be found here [43].

**Support Vector Machines (SVM)**[44], [45] were the main classification algorithm before the advent of neural networks and could be applied to quite complex types and high-dimensional data [46]. Unlike k-NN, the SVM method constructs a plane that separates the objects of two classes. The equation of this plane in feature space can be used as a criterion for determining whether an object belongs to one class or another, a simple example of building a classification criterion is shown in Fig. 1 (d). Like other classification algorithms, it is also commonly used in medical applications [47]. However, SVM has a few major weaknesses: lack of probabilistic interpretation, and sensitivity to noisy and missing data. Also, SVMs are inherently binary classifiers and do not directly support multi-class classification. While several strategies exist to extend SVMs to handle multi-class problems (e.g., one-vs-one, one-vs-all), these approaches can be less efficient and may require additional computational resources.

The formation of large data sets, as well as the need for their storage, exchange between medical institutions, structuring, and processing, have created prerequisites for their analysis with the aim of identifying patterns and forming working hypotheses. Hypothesis or feature generation in the case of classical ML still remained a human responsibility zone. Despite such an apparent disadvantage, classical ML models occupy their niche as they have a number of advantages compared to more advanced deep learning (DL) models. One of the main advantages of ML models is their sufficiency in a small amount of data for model building, which is often the key quality, for example, in medicine or medical research using AI models, the main problem is precisely the small database size. Secondly, ML methods do not require significant computational resources and relatively fast model training times. The third property is both an advantage and a disadvantage; for ML methods - manual feature indication is required for model training. This approach requires more developer involvement and effort to find these features, but a strong advantage of this approach is the interpretability of the model results, which is lacking in DL models. However, on large volumes or complex data, such as images, DL models significantly outperform ML models.

### 3.1 Deep learning

**Figure 2:**
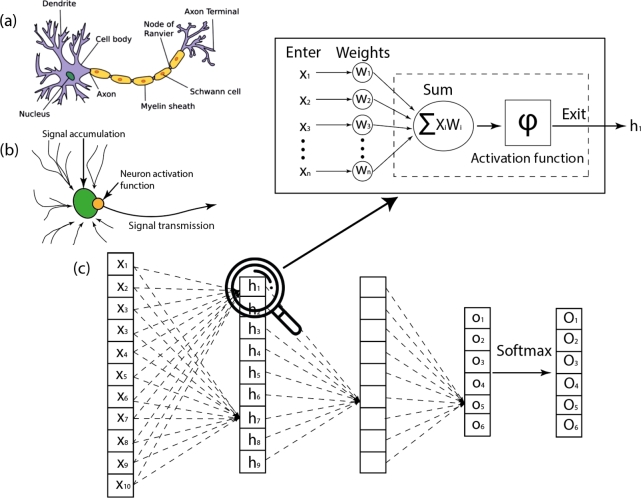
(a) Neuron (b) Illustration of signal flow through a neuron (c) MLP neural network composed of layers of a simple neuron model.

The most intricate form of ML is Deep Learning, a type of ML that encompasses neural networks. A basic example is illustrated in Fig. 2, designed for modeling multi-level abstractions from multiple data processing layers [48].

The concept of using a certain amount of data to train algorithms isn't novel. The first studies on this subject were published about 50 years ago [49], At that time, the perceptron model was introduced, drawing analogies from the functioning of a biological neuron. Further advancements were made in the 1980s [50], [51]; however, they didn't gain the widespread recognition that they have today and remained a subject of research, rather than applications, due to various reasons. Firstly, there wasn't adequate computational power available for the commercial application of ML models. Secondly, the data volumes were considerably limited. This situation paved the way for the development of other data analysis methodologies, notably mathematical statistics. The key and beneficial distinction from ML is the interpretability of this approach. This attribute is critical for many industries, including medicine, where statistical models are still in use today.

DL is a subsection of ML. Its primary distinction from traditional ML is the automatic feature determination during the algorithm training process. For instance, instead of manually programming that an ST elevation of more than 1 mm corresponds to an ST-elevation myocardial infarction, DL models can autonomously recognize the ST segment as a significant characteristic, without human intervention, and use this for diagnosis prediction. This revolutionary enhancement in learning algorithms not only saves human time and effort but also minimizes the likelihood of decision-making errors. For example, DL has significantly advanced computer vision, allowing for the automatic study of visual features from images or video content to obtain diagnostic and prognostic information [52]. This has enabled the automation of image analysis and interpretation, such as computer tomography, magnetic resonance imaging, electrocardiograms, and echocardiography [53], [54], [55], [56].

Another key algorithmic feature of DL is the large number of layers in the neural network. In Fig. 2 (c) an MLP neural network is depicted with only three “hidden” layers (excluding the input and output layers). This is considered a very simple or small neural network. Yet, even such a network can outperform traditional methods. Each cell in such a layer represents a “neuron,” a term inherited from biology, where the inspiration for the first neural networks originated [49]. Fig. 2(a) schematically shows a typical neuron composed of a nucleus, neuron body, axons, etc. The signal transmission process from neuron to neuron is well understood. But, when heavily simplified, it can be represented as shown in Fig. 2(b). Broadly, this process can be divided into three parts: signal accumulation, activation of the nucleus function (or activation function), and signal transfer to subsequent neurons as in Fig. 2(c). Such a simple model can be mathematically represented as a basic neural network in Fig. 2(c) where each cell of the network depicts a simple nonlinear function. As it turns out, an ensemble of such neurons representing a complex nonlinear function can be trained to solve a vast array of tasks, thanks to modern methods of parameter optimization, or in other words, network weight optimization. Such a network can be utilized for all vectorized data types. Increasing the number of layers theoretically can achieve incredibly accurate results. However, an overtrained network might prove useless as it lacks generalization capability. This effect can be intuitively explained as the network memorizing the training dataset without capturing its essence. This problem can be addressed in several ways, for example, loss function regularization or specific network training techniques [57], [58], [59], But that's not the only issue. For instance, neural networks are resource-intensive, consuming vast computational power. There's also the challenge of gradient vanishing (or, in other words, optimization adjustments) when training the neural network from layer to layer [60]. The problems of gradient vanishing and optimization of computational costs for training or neural network inference are addressed through neural network architecture optimization and its internal blocks. For each data type, various neural network architectures exist. In computer vision, the foundational ones are convolutional neural networks (CNNs).

### 3.2 Convolutional neural networks

**Figure 3:**
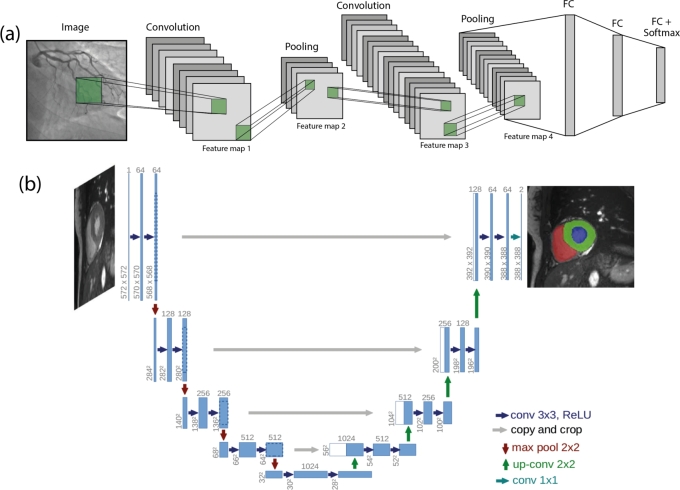
(a) A simple example of a CNN composed of convoluted blocks. (b) U-net architecture (example for 32x32 pixels in the lowest resolution). Each blue box corresponds to a multi-channel feature map. The number of channels is denoted on top of the box. The x-y-size is provided at the lower left edge of the box. White boxes represent copied feature maps. The arrows denote the different operations.

Computer vision evolved long before it became widely known. For instance, as early as the 1980s, a computer vision algorithm based on CNN layers could recognize handwritten digits [51]. However, the widespread adoption and development of CV came about in recent times thanks to increased computational capabilities. A convolutional layer is a fundamental building block for the entire field of computer vision. A colored image is input into the convolutional layer in RGB format, meaning as a tensor comprising three matrices (three layers of standard matrices), where each matrix contains the brightness of red, green, and blue colors. For black-and-white images, a single matrix suffices, as shown in Fig. 3 (a). The convolutional layer consists of a set of filters applied to the input signal (data). The output from the layer is a tensor made up of convolution results for each filter on the tensor layer. These filters are matrices (or in other terms, convolutional kernels), where their values are convolution parameters that are trained (determined) using optimization methods. The resulting tensor is often referred to as a feature map. Besides the fact that the convolution operation is less costly for image processing and offers translational invariance, which MLPs lack, this is critically important in classification tasks. Simply put, this property ensures consistent recognition results for shifted images. A breakthrough in classification tasks occurred in 2012 [61], which demonstrated the significant advantages of such models. Since then, models have been improved, with an increased number of layers and modified architectures. For instance, to address the problem of gradient vanishing during the training of large models, the ResNet model was proposed [62]. One of the most critical innovations was the use of skip connections. These connections pass gradients through network blocks, enabling much improved training of deep neural network layers.

DL systems can assist doctors by offering a second opinion and highlighting important features in incoming data, be it X-ray analysis [63], [64], [65], blood analysis [66], ECG [67] or even psychological patient analysis [68]. Currently, CV is actively permeating every corner of medical science. This is facilitated by the nature of the field, where images or videos are one of the most important sources of information. Object classification [69], object detection [70] and segmentation [71], [72] are key areas. Classification tasks are among the most common and were among the first to employ DL algorithms; an essential application of such algorithms is rapid patient screening. Another crucial task is image segmentation, where the U-net 3(b) [73] remains the most popular architecture, originally designed for biomedical image analysis. Today, such models have gained widespread use, for instance, in detecting atherosclerotic plaques in vessels [74] or assessing the size of various types of tumors [75], [76]. At present, deep learning models have achieved the accuracy of physicians and, in some cases, surpass them in a wide range of diagnostic tasks, including but not limited to differentiating a mole from melanoma [77], diagnosing diabetic retinopathy [78], determining cardiovascular risk [79] [80] and more. However, despite these impressive results, the practical application of AI is still limited due to its “black box” nature. More detailed issues regarding AI implementation are covered in the subsequent section. There are other AI applications where the cost of error is low, but time-saving can be significant, such as text processing.

**Natural language processing** Natural language processing is an AI field constructed to work with text data. This became particularly relevant with the advent of large language models and applications like ChatGPT [81]. Thanks to such models, interacting with documents and clinical records for quick searches or information accumulation became possible, potentially reducing time spent reading all documents significantly. Neural networks are used not only for diagnosis or aiding the treating physician but are also becoming prevalent in pharmacology, for instance, in determining molecular properties or drug design [82]. Fast creation or finding a suitable drug for a patient would pave the way for personalized medicine. An example of using AI to predict molecular properties is graph neural networks [83], where a molecule is represented as a graph and input into the neural network.

## 4 Machine learning algorithms in interventional cardiology

The rapid growth of endovascular procedures, as well as the introduction of new methods for non-invasive and invasive diagnosis of cardiovascular diseases, have promoted interventional cardiology as one of the main areas of implementation of artificial intelligence technologies. For example, it has become possible to simulate real-time fractional flow reserve (FFR) values based on computer tomography (CT) angiography data using artificial intelligence (instead of invasive FFR measurements), which can significantly expedite the interpretation of invasive coronary angiography results [84]. Applied to coronary angiography, this may accelerate the procedure, reduce radiation exposure, and help avoid possible complications associated with invasive interventions. Another example is the analysis of coronary stenosis during diagnostic coronary angiography, one of the most commonly performed interventional cardiac procedures worldwide, which is usually performed using visual assessment. Due to this, the method suffers from high operator dependence and poor reproducibility [85], [86], [87], [88], [89]. However, quantitative coronary angiography is capable of providing reliable quantitative measurements on coronary angiograms [90], [91] and is considered as the gold standard for stenosis assessment. DL algorithms can now perform all tasks required for the automatic interpretation of coronary angiograms, such as left/right coronary artery identification, anatomy description, vessel segmentation, stenosis localization, and stenosis severity prediction, leading to reduced variability and higher standardization of diagnostic angiograms [92], [93]. One of the application points of neural networks is the automation of data analysis processes, which eliminates operator error and also reduces the time required for analysis. As an example, the analysis of intravascular ultrasound data using neural networks can be mentioned. Nowadays, manual delineation of the coronary artery lumen and media boundary zone is not required, as this process is automatically performed by the neural network upon activation of the corresponding command. Simultaneously, the media-media diameter, residual lumen diameter, calculation of the residual lumen area, and the total vessel area are determined with minimal time expenditure [94]. If needed, virtual histology of the atherosclerotic plaque can be obtained, allowing for the identification and quantification of its histological components [95]. By leveraging neural networks, it is also possible to automate the differentiation of stable and unstable atherosclerotic plaques during intravascular ultrasound examinations [96], [97].

### 4.1 AI in intravascular imaging techniques

Intravascular imaging techniques, such as optical coherence tomography (OCT) and intravascular ultrasound, now play a key role in achieving optimal results of PCI. These techniques provide information about the anatomical and morphological characteristics of atherosclerotic lesions, allow the evaluation of stent implantation results (stent malapposition, presence of edge dissections, and degree of stent endothelialization), and facilitate the manipulation of coronary guidewires especially in patients with complex coronary lesions (chronic total occlusions, bifurcations, left main disease). However, obtaining this information is associated with several challenges: firstly, image processing is time-consuming, since the analysis of a single stented segment can take between 6 to 12 hours. Second, data interpretation requires extensive experience in performing such procedures, which limits the reproducibility of the obtained results. Moreover, traditional approaches face considerable difficulties in accurately classifying and organizing the majority of the acquired data. The primary objective of intravascular visualization methods is the assessment of the morphological characteristics of the atherosclerotic plaque (AP) and the risk of its destabilization. According to research data, AP (which has a number of pathohistological features, such as a large lipid core, a thin fibrous cap, neovascularization, etc.) is responsible for the development of acute coronary syndrome. Precise identification of these components is essential for the timely prevention of acute cardiovascular events. Currently, a significant number of algorithms have been developed to target the identification of vascular wall structure and various components of AP [98], [99], [100], [101]. Thus, Chu et al., based on OCT data from 391 patients, developed software for analyzing the characteristics of AP, which was further validated on 300 OCT images from 3 independent laboratories [102]. In this case, consensus among all three laboratories in plaque assessments was taken as the gold standard. The developed model accurately identified 518 out of 598 plaque areas with a diagnostic accuracy of 97,6% for fibrous plaques, 90.5% for lipid plaques and 88.5% for calcification. The average time required for analysis was 21.4 (18.6–25.0) seconds per series of images. Based on the developed algorithm, Hong et al. analyzed OCT data from 604 patients with myocardial infarction and non-target vessel stenosis and evaluated the prognostic value of the obtained data concerning adverse events. The authors also proposed new morphological (lipid-to-cap ratio) and physiological (optical flow coefficient) indices and compared them with traditional parameters (thin-layer AP and minimum lumen area) [103]. Both indices demonstrated superiority over traditional parameters in predicting adverse events related to the target vessel.

In another series of studies, Liu et al. developed an algorithm for the automatic detection and quantitative assessment of calcification using IVUS data and evaluated its prognostic impact on long-term clinical outcomes. The established model was tested on 35 image series obtained using 3 different IVUS systems [104]. The cross-validation accuracy for each system was > 0.9, with the model's final accuracy being 0.87, 0.89, and 0.89 for all three systems, respectively. The prognostic value of the algorithm was evaluated in a cohort of 408 patients [105]. The calcium index, calculated using the algorithm, was associated with adverse cardiovascular events up to 6 years (OR for calcium index >85 was 1.51, 95% CI 1.05–2.17, p-value = 0.026). Interesting findings were obtained in a study by Shibutani et al. [101]. The study evaluated histological samples from 45 autopsy hearts for comparison with ex vivo OCT images. The images were segmented and classified into four histological categories: pathological intimal thickening, fibroatheroma, fibrocalcified plaque, and healed erosion/rupture. The model consists of three components, namely the encoder, pyramid pool, and decoder module. When presented with an input image, the encoder module receives a feature map. Afterwards, the pyramid pooling module combines feature maps from different pyramid levels, followed by up-sampling and concatenation levels to form the final object representation. Finally, in the decoder module, a convolutional layer generates the final output image. The overall agreement rates with the histopathological diagnosis as the gold standard were 75% (*κ* value = 0.65) and 77% (*κ* value = 0.67) in the creating and validation groups, respectively.

In order to automate the analysis of “unstable” APs, it is crucial to accurately evaluate all of their components. Thus, in their work, Liu et al. proposed a comprehensive algorithm for the automatic detection of “unstable” APs using OCT data [106]. The algorithm consists of four modules: pre-processing using noise reduction and data augmentation methods, detection of “unstable” atherosclerotic plaques and their components using CNNs, post-processing, and aggregation of results obtained from different networks. When evaluating the algorithm on 300 OCT images, the model demonstrated a precision of 88.84% and recall of 95.02%. In the study, Rui Lv et al. proposed novel quantitative indices characterizing the “instability” of APs: the fibrous cap thickness index and the stress/strain index. The authors utilized ML methods, specifically random forest, to predict changes in the obtained indices based on combined OCT and IVUS data. The accuracy of the predictions for the obtained indices was more than 83.3% [107]. Liu and colleagues proposed an innovative approach for detecting thin-cap fibroatheromas [108]. The authors developed a multi-view contour-constrained transformer network, which effectively captures distinctive features from the region of interest, specifically the vascular wall. The main feature of this approach is the utilization of transformers [109] and the assumption that providing two images from different perspectives (polar and cartesian) should complement each other and enhance the prediction quality of the neural network. The proposed algorithm demonstrated advantages over other methods that utilize one or multiple perspectives on a publicly available (semi-public) dataset. However, further validation is required to confirm its effectiveness.

**Figure 4:**
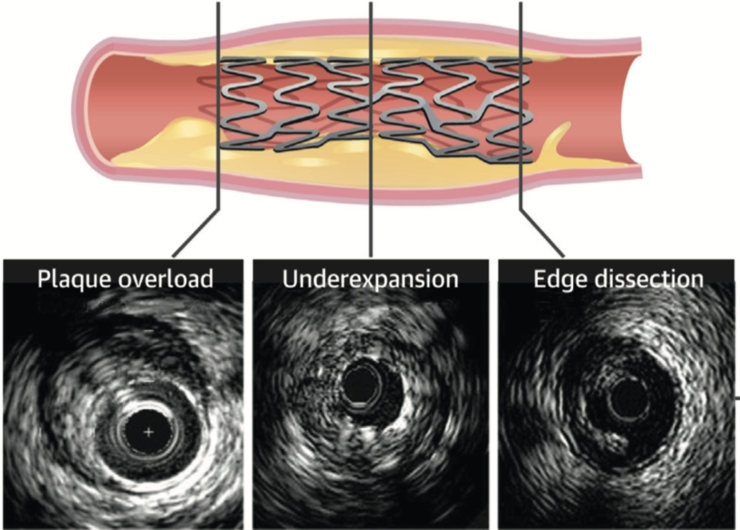
Suboptimal characteristics of stented segment analyzed using IVUS [110].

Another direction in the development of intravascular visualization is focused on assessing the outcomes of stent implantation and detecting suboptimal results that require further optimization. The most frequent IVUS findings are shown in Fig. 4. Currently, several algorithms have been developed to automatically detect stent struts, vessel lumen, and the degree of endothelialization [111], [112], [113] Most of the algorithms are primarily based on analyzing OCT images, which provide higher resolution compared to IVUS. Thus, based on OCT data, the software has been developed to automatically detect vessel lumen, assess the degree of stent strut endothelialization, and identify stent malapposition. In comparison to manual analysis, the software achieved a correct classification rate of 82% for uncovered struts and 99% for endothelialized struts. It is important to note that the average analysis time for a single image series was 27±18 minutes, which is significantly faster compared to manual analysis. Additionally, the speed of analysis was dependent on the quality of the imaging. Indeed, images with a higher amount of blood required over 1 hour for analysis. Certain difficulties in the analysis may arise in patients with extensive stent endothelialization, resulting in a weaker signal from the stent struts. In addition, patients with multiple layers of stents in the vessels can pose challenges during analysis. In their study, Yang et al. developed an algorithm for automatic analysis of stents, both with thin layers (<3 mm) and thick layers of endothelium (>3 mm), including patients with multiple layers of stents. The algorithm was developed based on data from 41 patients [114]. The accuracy of the algorithm was 0.932±0.009, with a sensitivity of 0.939±0.007 for stents with endothelialization thickness ≤ 0.3 mm, and 0.856±0.019 with a sensitivity of 0.874±0.011 for stents with endothelialization thickness > 0.3 mm. A more comprehensive approach was proposed in the study by Wu et al., where a convolutional model was developed for automatic detection and segmentation of stent struts [115]. By incorporating pseudo-3D images, the study by Wu et al. was able to combine information from adjacent frames, resulting in improved accuracy in stent strut detection and segmentation. After training, the model was tested on 21,363 OCT images and demonstrated excellent segmentation performance (0.907 DICE) and stent strut detection accuracy (0.943 precision). The average time required for three-dimensional reconstruction of a single series of images was 9.22±2.82 seconds. Moreover, there are emerging studies where developed algorithms for assessing intravascular imaging are being used for predicting procedural and clinical outcomes. Indeed, in a recent study by Min et al., a model was developed based on the analysis of IVUS data from 618 patients to predict final stent under-expansion. The model exhibited excellent discriminative ability, with an area under the curve of 0.94 [116]. First, of its kind, Hamana et al. developed an algorithm that utilizes OCT image processing data to predict the risk of target lesion failure. To pre-train the model, a total of 162,523 images were analyzed using self-supervised learning methods to create a planner CNN capable of efficiently extracting specific features from OCT images. The features extracted from the trained model were fed into a spatial CNN and transformed into hazard probabilities for each survival interval. The c-index for the model in the validation group was reported as 0.762 [117]

### 4.2 AI based navigation in interventional cardiology

**Figure 5:**
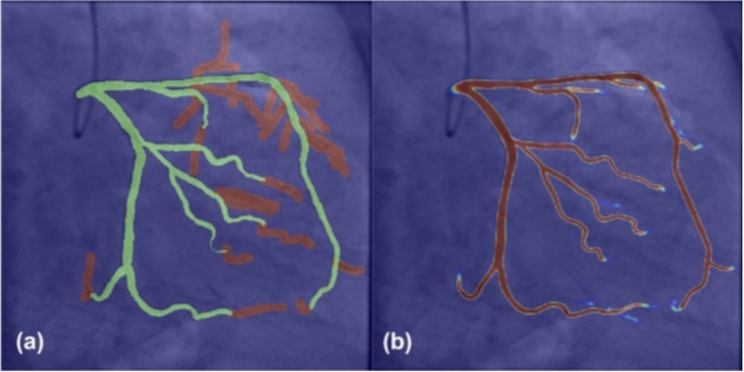
(a) Annotations of large (green) and small vessels (red), (b) Overlay of the input frame and the predicted probability map. [119].

Furthermore, advancements in ML technologies have significantly advanced the performance of endovascular procedures through the introduction of new navigation systems that provide real-time dynamic visualization of coronary arteries without the need for contrast agents [118]. In this regard, based on the existing coronary angiogram, the software generates a digital overlay with compensation for respiratory and cardiac motion, which is subsequently superimposed on the fluoroscopic image as shown in Fig. 5.

**Figure 6:**
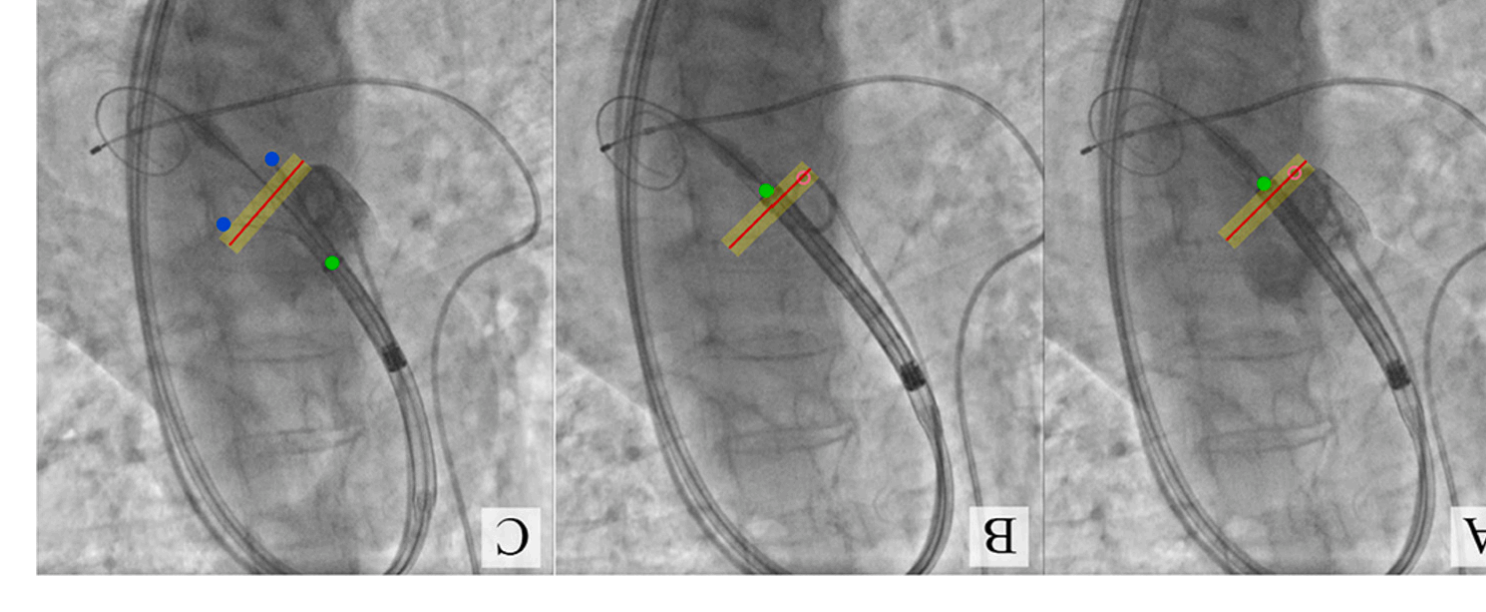
(A) Target implantation site tracked by the pigtail location during the contrast injection; (B) tracking of catheter location relative to the aortic annulus plane with an acceptable implantation error in the absence of contrast; (C) imaging with partial extraction of the valve from the delivery system. [121].

Indeed, in the study by Piayda et al., it was demonstrated that this technology provides an anatomically accurate overlay of coronary vessels with satisfactory image quality in 98% of cases [120]. Several studies have shown that dynamic coronary mapping technology, compared to the traditional approach using contrast injections, is associated with a significant reduction in contrast agent volume (22%) and fluoroscopy time (30%), despite similar clinical and procedural characteristics. Another category of endovascular interventions that require high operator accuracy is transcatheter aortic valve implantation. Indeed, the optimal implantation zone for the transcatheter valve is approximately 1 cm, and displacement of the prosthesis below or above this level can result in complications such as atrioventricular block or prosthesis dislocation into the ascending aorta. The currently available anatomical models, created preoperatively using computed tomography, angiography, and other imaging techniques [121], do not provide a visualization of the dynamic changes in the aortic lumen during valve implantation as they are static representations. To overcome these limitations, a navigation system has been proposed that is based on tracking 11 key points in the aortic root and valve delivery system, allowing the operator to intraoperatively identify any deviation from the optimal trajectory [121]. A potential application of this approach is the real-time display of a valve implantation zone indicator, enabling optimal navigation during the procedure (Fig. 6).

Of course, such technologies can have a significant impact on the effectiveness of the procedure and patient outcomes, as well as reduce the negative effects of radiation on both medical personnel and patients.

### 4.3 Coronary artery segmentation

Coronary artery segmentation plays a fundamental role in the implementation of AI in interventional cardiology. This is because in order to solve such tasks as identification, classification, calculation of physiological indices, and virtual stenting, it is necessary to distinguish the object of interest (coronary arteries) from irrelevant information (bone structures, thoracic organs). This process is called segmentation, and the quality of the constructed model depends on the initial quality of angiographic images, which, in turn, is influenced by various factors such as cardiac motion, presence of X-ray-absorbing tissues (ribs, vertebrae, etc.), low signal-to-noise ratio, presence of low-contrast regions, degree of contrast agent filling in the coronary arteries, patient weight, and many others. All these factors contribute to the challenges in obtaining accurate models of the coronary vessels, which can subsequently lead to incorrect diagnosis and determination of an inappropriate treatment approach. The CNN model with an encoder-decoder architecture remains the best choice for segmentation tasks. The encoder extracts features from the image and converts the input image into low-resolution object maps, while the decoder projects the learned data onto the original images with pixel-wise predictions. The performance of segmentation is predominantly evaluated using metrics such as DICE coefficient and Jaccard index, which reflect the regional overlap between the generated model and the ground truth object. Currently, segmentation techniques are applied to both the general outlines of vessels obtained from coronary angiography and the lumen of the arteries based on intravascular imaging data.

**Figure 7:**
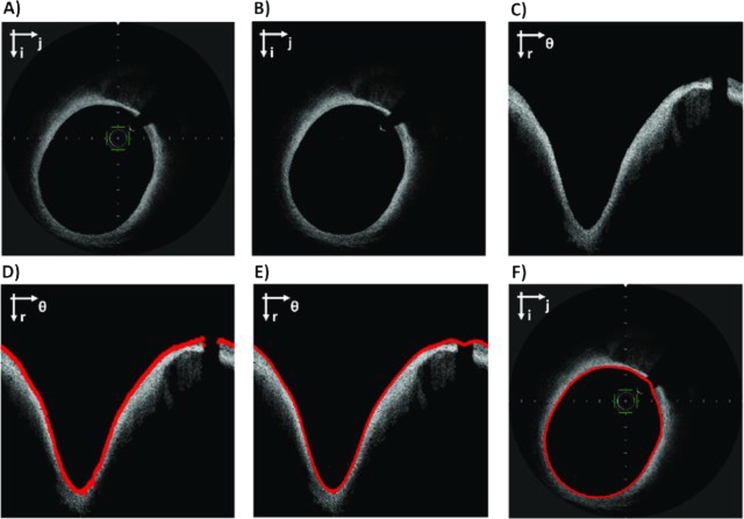
(A) Original OCT image in Cartesian coordinates. (B) Image after pre-processing. (C) Image in polar coordinates. (D) Raw lumen contour detection (red). (E) Lumen contour after interpolation (red). (F) The original image in Cartesian coordinates with the segmented lumen contour (red). The Cartesian coordinate system (i; j) or the polar coordinate system (r; *θ*) is indicated on the top left of each image. [124].

The segmentation of artery lumen is considered to be one of the relatively simple tasks for ML methods, especially in the absence of atherosclerotic lesions and with high-quality input images. For this purpose, binaryization methods such as Otsu filtering, edge detection, and curve fitting [122], [123], [124], [125], [126] are commonly used. The workflow of lumen is segmentation presented in Fig. 7. Nonetheless, these methods exhibit lower efficacy when segmenting bifurcation lesions, in the presence of artifacts from the catheter or guidewire, and when the catheter lumen is not completely cleared of blood during imaging, which are common challenges in everyday clinical practice. Several approaches have been proposed to address these challenges, aiming to improve the segmentation accuracy in such cases. These approaches have shown good agreement with expert annotations [127]. However, the effectiveness of these approaches can decrease in patients with more complex lumen geometry, challenging bifurcations, and stented segments. To improve the ability to classify and segment the lumen in complex coronary lesions such as stented arteries and bifurcations, numerous approaches have been proposed. Indeed, to overcome lumen contour disruptions caused by bifurcations, Akbar et al. proposed an interpolation-based approach using analysis of cross-sectional (C-mode) and longitudinal (L-mode) OCT images [128]. The lumen contours extracted using the proposed scheme are aligned to form a three-dimensional (3D) coronary geometry of the depicted artery. Applied to 5931 images (40 patients), this approach demonstrated a strong correlation between manual and automatic segmentation (R = 0.98). In the study by Miyagawa et al., interesting findings were obtained as they compared four different CNN architectures: the baseline network using stochastic gradient descent, and three networks utilizing transfer learning from a previously trained network [129]. The area under the curve for the obtained model was 99.72 ± 0.17, which outperformed other bifurcation classifiers [130], [131]. Moreover, there was no statistically significant difference found between the results using images in the polar and Cartesian coordinate systems, eliminating the need for preprocessing the images into polar form. In another study, Yang et al. compared the effectiveness of six classifiers (RF, SVM, J48, Bagging, Naïve Bayes, and AdaBoost) in challenging lesions of the arterial lumen [132]. By identifying and classifying 92 features from 54 patients and 14,207 images, the RF classifier demonstrated the highest accuracy compared to the other five classifiers (RF 98.2%, SVM 98.1%, J48 97.3%, Bagging 96.6%, Naïve Bayes 88.8%, AdaBoost 88.7%) However, residual blood artifacts and clots decreased the accuracy of segmentation, which was subsequently improved by Yong et al. using a linear regression CNN trained on a dataset of 19,027 images [133]. It should be noted that as models become more complex, detailed information can gradually be lost due to decreased resolution, making classification and segmentation accuracy more challenging. To address this issue, Tang et al. proposed a solution by introducing a new CNN based on N-Net, which can reuse the original input image in deeper convolutions to connect high-resolution input data with low-resolution feature information [134]. The proposed model was trained on 20,000 images and demonstrated excellent agreement with expert annotations, even in the presence of complex lumen anatomy (accuracy: 0.98 ± 0.00; specificity: 99.40 ± 0.05; DICE: 0.93 ± 0.00). N-Net also resulted in significantly lower losses (0.08) compared to traditional U-Net architectures (0.11-0.15). Such approaches can help in the accurate and efficient creation of three-dimensional lumen geometries for the assessment of quantitative flow reserve or local hemodynamics almost in real-time [135], [136].

Given that selective coronary angiography is a primary method used in both the diagnosis and treatment of coronary artery disease, the development of technologies that enable real-time generation of coronary artery models is of paramount importance in improving the outcomes of coronary artery disease treatment. To date, several studies have been published on the segmentation of coronary arteries using AI technologies. For instance, Yang et al. successfully developed a model for the segmentation of coronary arteries based on 3,302 coronary angiograms and demonstrated that real-time prediction can be achieved with minimal preprocessing of the image [137]. The average F1 score reached 0.917, and 93.7% of the images had a high F1 score > 0.8. The most stenotic area was clearly captured with high coherence. However, it is important to note that in this study, only specific segments of major arteries were segmented, without including the side branches.

It is important to note that potential technical challenges arise in the analysis of coronary angiograms due to the mismatch between the two-dimensional X-ray angiography images and the actual 3D vascular anatomy. Physicians primarily address this discrepancy through intuition and tactile feedback, which can lead to potential errors and high operator dependence during the procedure. Although 2D X-ray images allow for the identification of vascular motion in real-time, they remain two-dimensional, resulting in a loss of some information about vessel anatomy. The technical limitations of 3D computed tomography lie in the fact that these images are acquired at the end of diastole or systole; thus, there are constraints in representing the motion of blood vessels in real time. The use of neural networks may allow a synthesis of these two studies and create a 3D model of the vascular bed in real time. Indeed, Taeyong Park et al. conducted a study based on a novel segmentation method using a CNN and non-rigid registration, which involved analyzing the phase structure and comparing similar vascular elements on 3D CT images obtained prior to the procedure and 2D angiographic images acquired during the procedure from the same patient [138]. A CNN was used for the segmentation of coronary arteries in 2D angiographic images in real-time, acquired from various projections. To compensate for errors in the 2D angiographic imaging process, 3D computed tomography (CT) was used for analyzing the topological structure of the coronary tree. The authors evaluated the proposed method on 50 observation series from 38 patients, comparing the results with ground truth. According to the experimental results, the error for all vessels was 0.8705 mm, the error for a manually placed marker was 1.06 mm, and the error for the bifurcation point was 1.5706 mm. Moreover, the overall segmentation time was 0.179 seconds. In another major study conducted by Du et al., the focus was on two tasks: coronary artery segmentation and identification of lesion morphology (calcification, stenosis, thrombus) [93]. For the first task, a dataset of 13,373 annotated coronary angiograms was used. To address the task of coronary artery recognition, the authors modified a conditional generative adversarial network (cGAN) for image segmentation. For the task of lesion morphology detection, a convolutional deep neural network (DNN) was developed, which outputs the locations of various lesions appearing in the input angiogram. For each input angiogram, the algorithm combines the outputs of the coronary artery recognition DNN and the lesion detection DNN to create high-level diagnostic information, including the identification of each coronary artery lesion and the segment of the coronary artery where it is located. For the segmentation prediction, the recognition accuracy was 98.4%, and the recognition sensitivity was 85.2%. For the detection of lesion morphology, including stenosis, total occlusion, calcification, thrombosis, and dissection, the F1 scores were 0.829, 0.810, 0.802, 0.823, and 0.854, respectively. The recognition time was 2 seconds. In the study by Menezes et al., not only was a model developed for coronary artery segmentation, but specific criteria were also proposed to assess the quality of the proposed model [139]. The segmentation was performed using a U-Net-based CNN, commonly used in medical image segmentation. The results of the initial model were further refined by expert specialists and served as the foundation for an improved model. The performance of the model was evaluated using both well-established methods and the proposed criteria, which were combined to provide an overall assessment of the segmentation. The final model demonstrated a very high Generalized Dice Score (0.9348±0.0284), with higher performance in artery segmentation compared to catheter segmentation. The improved model achieved an average score of 90 based on a created scale, indicating that it provided 90% of what experts considered most important in coronary angiography analysis. Subsequently, the authors validated the developed model on 117 angiograms with at least one significant coronary artery stenosis marked [140] The accuracy of overlap, sensitivity, and DICE score between the ground truth and segmented coronary angiogram were 99.9%, 95.1%, and 94.8%, respectively. Furthermore, the severity of stenosis differed by less than 5% in absolute terms, which was statistically and clinically insignificant.

## 5 The challenges of implementing machine learning

Despite the active development of ML technologies and the multitude of algorithms that have been developed, their implementation in clinical practice is still limited. Firstly, this is due to the lack of large-scale datasets. Currently, the vast majority of medical records are designed for reporting purposes to insurance companies rather than for research [3]. Furthermore, restrictions on the use of personal data are also a major obstacle to extracting statistical information from archived data. Applying ML technology to analyze big data requires a high degree of standardization and integration of various data sources within and between hospitals, as well as access to previous patient data. Another challenge is the quality of the input data, as the quality of prediction models depends on it. With low-quality input data, there is a high risk that the resulting model will produce erroneous data, and overestimate or underestimate risks for individual patients, ultimately affecting the quality of treatment outcomes. Secondly, the data regarding the integration of developed models into clinical practice is limited [141], [142], [143]. Currently, most created models have demonstrated their efficacy during development but require further validation and comparison with traditional approaches in large-scale clinical studies. However, it is important to mention the issue of different methods for evaluating algorithms and traditional statistical methods of data analysis and their comparison. Applying standardized definitions for evaluating the obtained models, similar to those used in clinical research, can significantly expedite the integration of ML into various medical domains. A significant limitation, often overlooked in research, is the processing time of AI algorithms. Higher model accuracy often translates to more complex architecture and longer processing times [144]. Real-time application of artificial intelligence should not lead to delays that can impact patient outcomes. Therefore, there needs to be a trade-off between accuracy and speed in deployed models.

Another challenge of ML methods, especially complex DL models (neural networks), is the difficulty in interpreting the results of these models, which are complex functions with millions of parameters. Such models make predictions based on the input data without providing a rationale behind the decision-making mechanisms. This lack of interpretability slows down the process of gaining new knowledge and hinders their practical implementation. However, specialized tools are emerging that can explain the mechanisms of operation of such complex deep learning models [145]. Therefore, it is important to understand that AI cannot replace an experienced physician but can certainly be a valuable assistant to them.

It is also important to consider the legal issues. For example, will physicians be legally protected if their treatment is based on AI applications, as the physician remains responsible for the life and health of the patient [146]. The safety and efficacy of such algorithms have already been addressed by the U.S. Food and Drug Administration and the European Union, which have issued regulatory guidelines for the deployment of AI, requiring pre-assessment before AI software can be used as a medical device [147].

### 5.1 Ready-to-use software

Despite the impressive integration of various visualization methods in interventional cardiology, they are still primarily limited to two-dimensional imaging. The fusion of images from different non-invasive visualization modalities, such as ultrasound, magnetic resonance imaging, OCT, and IVUS, is challenging to achieve, although significant progress has been made in this area in recent years [148]. The fusion of 3D reconstruction data based on CT with fluoroscopy is particularly useful in structural heart interventions and in the treatment of patients with chronic total occlusions that require clear visualization of the entire vessel [149]. The combination of angiographic images with IVUS, OCT, and physiological measurements is also widely employed, allowing for additional characterization of plaque morphology and assessment of lesion significance [150]. Currently, there are software packages available for the rapid integration of CT and MRI data with fluoroscopic images (e.g., VesselNavigator system by Philips Healthcare) [151]. This image overlay technology enables the 3D reconstruction of target structures and the integration of these images with fluoroscopy data, providing mapping for complex trans-catheter interventions that require high precision and are associated with high risk. The next step towards more realistic 3D/4D rendering and visualization could be holography [152]. However, this would require prospective reconstruction based on a pre-existing CT or MRI before the patient arrives in the interventional lab. The optimal approach to achieve this would be the real-time visualization of virtual and mixed reality [153]. A semi-transparent 3D hologram displayed in real-time in front of the interventional cardiologist has the potential to enhance procedural accuracy and reduce potential risks. These ideas have led to the emergence of a new interdisciplinary field known as “computer vision,” where artificial intelligence extracts a high level of understanding from digital images, similar to how a human does, and transforms it into a completely new model created at the intersection of various diagnostic methods. This technology utilizes multiple sources of information simultaneously, which is not possible for a human operator [154].

## 6 Conclusion

ML and DL methods are currently being comprehensively studied. However, it would be inaccurate to label the current level of achieved results as artificial general intelligence (AGI). At present computers act more as assistants to the doctor, saving time and performing routine tasks. Still, more progress is required before artificial intelligence can make independent decisions in medical practice. Having vast data and high computational speeds are not yet sufficient to create intelligent algorithms capable of predicting events. It's evident that there's always some degree of prediction inaccuracy due to the impossibility of collecting all necessary information about a process or phenomenon. Continuous biosignal monitoring (e.g., electrocardiogram, blood pressure, echocardiography, etc.) implies the emergence of a vast amount of real-time information. From a practical perspective, artificial intelligence will analyze radiographic and intravascular visualization data, ECG, vital signs in real-time, correlate them with previously obtained laboratory and anamnestic data, recreate computer models of a specific patient, and determine the preferred treatment method, and potential intervention risks, and predict key intervention moments. Medical oversight will decrease, limiting itself to differential diagnosis and choosing the most appropriate examination method, including the optimal data analysis algorithm. Big health data challenges traditional research methodologies and provides new ways to derive results from a vast number of sources. Further efforts are required to transition from the mere existence of big data to its widespread use, and better understanding of cardiovascular diseases. The potential of ML to enhance healthcare system efficiency is very high, but it necessitates extensive and lengthy work to integrate it into daily clinical practice.

## CRediT authorship contribution statement

**Dmitrii Khelimskii:** Writing – review & editing, Writing – original draft, Visualization, Project administration, Methodology, Investigation, Conceptualization. **Aram Badoyan:** Writing – original draft. **Oleg Krymcov:** Writing – original draft. **Aleksey Baranov:** Writing – original draft. **Serezha Manukian:** Writing – original draft. **Mikhail Lazarev:** Writing – review & editing, Writing – original draft, Visualization, Validation, Supervision, Project administration, Methodology, Investigation, Funding acquisition, Formal analysis, Conceptualization.

## Declaration of Competing Interest

The authors declare that they have no known competing financial interests or personal relationships that could have appeared to influence the work reported in this paper.

## Data Availability

No data was used for the research described in the article.
